# Supplementary material for: Using citizen science to determine if songbird nesting parameters fluctuate in synchrony
Source: PLoS One. 2022 Nov 16;17(11):e0277656. doi: 10.1371/journal.pone.0277656 (PMC9668184; doi:10.1371/journal.pone.0277656)
Supplement: S1 Table — Summary of random-effect model selection for (a) hatching success, (b) hatchability, and (c) fledging success of Eastern bluebirds and Carolina chickadees at the species and species-region levels. No fixed terms were included. Though the model with nest location was not always the most parsimonious, we included this random variable in all our models for consistency across analyses. Models are presented with corresponding k (number of parameters), AIC (Akaike’s Information Criterion), ΔAIC (difference in AIC between given model and lowest AIC model), and wi (model weight) values. (DOCX) [file pone.0277656.s001.docx]

| Level | Ecoregion | Species | Model | *k* | AIC | ΔAIC | *w_i_* |
| --- | --- | --- | --- | --- | --- | --- | --- |
| (a) Hatching success | | | | | | | |
| Species | All | Bluebird | Location | 2 | 2620.5 | 0.00 | 1.00 |
|  |  |  | Null | 1 | 2793.7 | 173.2 | < 0.001 |
|  | All | Chickadee | Location | 2 | 545.5 | 0.00 | 0.98 |
|  |  |  |  |  |  |  |  |
| Species-ecoregion | Atlantic Coast | Bluebird | Location | 2 | 981.0 | 0.00 | 0.99 |
|  |  |  | Null | 1 | 996.1 | 15.1 | < 0.001 |
|  | Atlantic Coast | Chickadee | Location | 2 | 187.3 | 0.00 | 1.00 |
|  |  |  | Null | 1 | 202.9 | 15.6 | < 0.001 |
|  | Central Hardwoods | Bluebird | Location | 2 | 1643.2 | 0.00 | 1.00 |
|  |  |  | Null | 1 | 1774.6 | 131.4 | < 0.001 |
|  | Central Hardwoods | Chickadee | Location | 2 | 342.1 | 0.00 | 0.91 |
|  |  |  | Null | 1 | 346.7 | 4.59 | 0.09 |
| (b) Hatchability | | | | | | | |
| Species | All | Bluebird | Location | 2 | 5181.6 | 0.00 | 1.00 |
|  |  |  | Null | 1 | 5267.7 | 86.1 | < 0.001 |
|  | All | Chickadee | Location | 2 | 1120.2 | 0.00 | 1.00 |
|  |  |  | Null | 1 | 1200.8 | 80.6 | < 0.001 |
| Species-ecoregion | Atlantic Coast | Bluebird | Location | 2 | 2157.1 | 0.00 | 1.00 |
|  |  |  | Null | 1 | 2212.8 | 55.6 | < 0.001 |
|  | Atlantic Coast | Chickadee | Location | 2 | 572.3 | 0.00 | 1.00 |
|  |  |  | Null | 1 | 598.5 | 26.2 | < 0.001 |
|  | Central Hardwoods | Bluebird | Location | 2 | 3014.0 | 0.00 | 1.00 |
|  |  |  | Null | 1 | 3045.7 | 31.7 | < 0.001 |
|  | Central Hardwoods | Chickadee | Location | 2 | 547.8 | 0.00 | 1.00 |
|  |  |  | Null | 1 | 586.4 | 38.7 | < 0.001 |
| (c) Fledging success | | | | | | | |
| Species | All | Bluebird | Location | 2 | 1135.3 | 0.00 | 0.88 |
|  |  |  | Null | 1 | 1139.3 | 4.04 | 0.12 |
|  | All | Chickadee | Location | 2 | 412.0 | 0.00 | 0.55 |
|  |  |  | Null | 1 | 412.5 | 0.41 | 0.45 |
| Species-ecoregion | Atlantic Coast | Bluebird | Location | 2 | 536.7 | 0.00 | 0.91 |
|  |  |  | Null | 1 | 541.2 | 4.51 | 0.09 |
|  | Atlantic Coast | Chickadee | Location | 2 | 138.8 | 0.00 | 1.00 |
|  |  |  | Null | 1 | 180.9 | 42.1 | < 0.001 |
|  | Central Hardwoods | Bluebird | Location | 2 | 600.0 | 0.00 | 0.51 |
|  |  |  | Null | 1 | 600.1 | 0.07 | 0.49 |
|  | Central Hardwoods | Chickadee | Null | 1 | 233.3 | 0.00 | 0.66 |
|  |  |  | Location | 2 | 234.6 | 1.34 | 0.34 |
