# Supplementary material for: Using citizen science to determine if songbird nesting parameters fluctuate in synchrony
Source: PLoS One. 2022 Nov 16;17(11):e0277656. doi: 10.1371/journal.pone.0277656 (PMC9668184; doi:10.1371/journal.pone.0277656)
Supplement: Attachment — Submitted filename: PONE-D-22-01496 Review.pdf [file pone.0277656.s002.pdf]

PONE-D-22-01496 Review

The authors conducted a new and interesting study on if synchrony of demographic parameters exists within and across populations of two bird species using citizen/community science data. The authors demonstrated strength in describing their methods and clearly stating their results. My main concern with the manuscript is that the authors did not explain why a broad-scale analysis of synchrony should be studied and that they centered their manuscript on using the citizen/community science dataset rather than how synchrony could affect bird populations. The authors need to refocus the manuscript on the importance of synchrony as it pertains to bird conservation and why it is important that we should examine synchrony across multiple populations rather than look at demographic parameters of individual populations. The conclusions of the manuscript are difficult to discern; the majority of the discussion focuses on gaps in the dataset or alternative explanations as to why few significant results were found. The authors need to provide broader context for increased synchrony in hatchability and how that could be a benefit under changing environmental conditions.

Major Revisions

<u>Abstract</u>

Lines 22-25: I would start your abstract introducing what synchrony is and how climate change is affecting it rather than stating the benefits of citizen science. You state that no study has examined synchrony across populations and species but you do not define what synchrony is or why it is important.

Lines 26-27: Please state the name (common and scientific) of the species you analyzed. You also need to state that hatching success, hatchability, and fledging success are the breeding parameters you measured; you use the term breeding parameters in the next sentence but you need to state that this term relates to the dependent variables mentioned above.

Lines 28-31: Your conclusions could use some more support; why would breeding parameters be buffered for temporal variation? Are there any other alternative explanations as to why synchrony was not found between populations? You transition into the factors that could have affected your study rather than focusing on what your conclusions say.

<u>Introduction</u>

Lines 36-37: Your first sentence has a strange transition. You could combine the first two sentences of your introduction to get the point across that long-term and large-scale data are needed to determine how environmental factors and climate change could affect species' demographic parameters.

Line 41: Any songbird examples of the subpopulation or population level not providing adequate information on species responses to change? While the penguin example is good, it is more relatable to have a songbird example since that is what you are studying. Also, the scientific name needs to be in parentheses.

Lines 45-86: I would restructure these paragraphs so that your synchrony paragraphs (54-86) goes before the citizen/community science paragraph (45-53). While the citizen/community science program is important and puts an emphasis on using these dataset in large-scale studies, the main focus of your study is how synchrony affects demographics of species and populations.

Lines 65-66: State what specific breeding parameters may be synchronized across populations and similar species.

Lines 87-89: Revise sentence to “To examine how breeding parameters between and within two southern cavity-nesting bird species respond to climate change and other changing environmental factors, we used 21 years of community science nesting data from The Cornell Lab of Ornithology’s NestWatch Program.” or something similar.

<u>Discussion</u>

Lines 251-253: You restate your question which is good, but you need to clearly state your conclusions, which I believe begin on line 256.

Line 254: State what these negative consequences could be.

Lines 268-269: From what I understand hatchability synchrony increased significantly over the past 20 years. You then spend the next few paragraphs discussing gaps in data, examining other species, and the benefits of citizen/community science projects. You need to expand on this result that hatchability synchrony has increased for bluebirds, and what the ramifications are in the context of environmental change and/or climate change.

Minor Revisions

<u>Introduction</u>

Line 73: Avoid starting sentences with “because.” You could use the word “since” instead.

<u>Methods</u>

Lines 103-105: Revise sentences so there is similarity between sentence clauses.

Line 125: After stating the ecoregions, put abbreviations in parentheses after. You use these abbreviations in Table 1; they should be used throughout the rest of the manuscript.

Line 163-164. Consider adding an Equation section at the end of the manuscript and having an Equation 1 label here. You also need to state what each of the variables mean in this equation.
