# Supplementary material for: Using citizen science to determine if songbird nesting parameters fluctuate in synchrony
Source: PLoS One. 2022 Nov 16;17(11):e0277656. doi: 10.1371/journal.pone.0277656 (PMC9668184; doi:10.1371/journal.pone.0277656)
Supplement: Attachment — Submitted filename: Plos One review March 2022.docx [file pone.0277656.s003.docx]

PLOS One review March 2022

Using citizen science to determine if songbird breeding parameters fluctuate in synchrony

This paper uses data collected from long term nest recording studies to examine synchronous fluctuations in breeding parameters of two cavity-nesting bird species and populations breeding in different areas of the eastern United States. The authors aim to assess the level of synchrony in breeding parameters between two species each of which have two populations, and to review the benefits of using citizen science data for addressing ecological questions. The authors’ analysis led them to conclude that there is not much evidence of synchrony in selected breeding parameters between species and populations, although due to limitations in the data it is not possible to conclude whether this is due to lack of synchrony or lack of appropriate data. The paper is well written in general, though there are major problems with study design and analysis that makes it difficult to draw conclusions from this work.

Examining synchrony among populations and species is a useful way of understanding how populations and species react to changes in the environment or climate. Synchronous changes in breeding parameters by species or populations suggest reliance on similar habitats or climatic conditions making certain groups of species vulnerable to environmental or climatic change. Although the aim of detecting synchrony in species/populations is useful, it is not clear in this study why these particular species/populations were chosen for comparison, and why attempts were made to detect synchrony in the selected breeding parameters. The paper could be improved by explaining more clearly in the Introduction the purpose of the study in terms of the species/populations/breeding parameters chosen and how the findings contribute to the field of ecology or conservation management.

The authors state in the Abstract that “no studies have used citizen science data to determine if demographic parameters fluctuate in synchrony across populations and species”. The issues with this statement are 1) that the definition of “citizen science” is unclear and 2) that many studies have been published that use data routinely collected by amateur ornithologists or reserve wardens to examine synchrony in demographic parameters across populations and species (e.g. Robertson et al. 2015). Also, one of the study aims stated in the Introduction is to “review the benefits and constraints of using citizen science data for assessing long-term and large-scale ecological questions.” I don’t think this has been adequately covered in the paper. The Discussion does include some discussion about the use of citizen science data, but more discussion could be included on 1) the definition of citizen science data; 2) how widespread the use of data collected by amateur scientists is in studies; 3) the advantages/disadvantages of using these data.

This study may also benefit from an examination of causes of synchrony between species, perhaps using weather/climate data. The analysis as it currently stands just looks for synchrony between species/populations at different time scales. Including an analysis of environmental/weather data that may drive potential synchrony would be interesting and would increase the impact of the study.

The Methods section states that GLMMs were used to estimate breeding parameters (lines 147-151), but the discussion of the results is missing in the rest of the paper. The authors could make it clearer why GLMMs were used in the analysis and how the results were used in the study.

**Minor comments:**

Lines 25-29: The authors do not make it clear why examining synchrony across these species is important. The abstract begins by focusing on the utility of citizen science, but fails to link this premise with the study aim of examining synchrony in demographics two cavity-nesting species.

Lines 25-29: Not clear here why the authors aimed to compare these two species - were the two species chosen simply because data were available or was there an underlying biological hypothesis driving this study? If so, make explicit here.

Line 29: “Breeding parameters could be buffered against temporal variability”; not clear what this phrase means.

Lines 47-48: The authors need to better define "large-scale scientific studies" and "citizen science programs". There may be some overlap between these (e.g. scientific studies may rely on data collected by volunteer amateur ornithologists, so not clear under these definitions whether these should be defined as “large-scale scientific studies” or “citizen science programs”)

Lines 51-53: It's not clear here what the authors mean by "citizen science data". Many studies have made use of data collected by ringers or bird watchers who were not directly involved in the study, but who collected data that were later used. For example, many studies have been published using long-term monitoring, collected by amateurs or reserve wardens and later analysed to examine synchrony among populations or species (see Robertson et al. 2015, Lahoz-Monfort et al. 2017).

Line 59-60: Could be clearer regarding which phenomenon the authors are referring to here.

Line 64: Add reference after “it can also be applied to closely related species”

Line 65: Add reference after “and species with similar life histories”

Line 65-66: Explain here what factors are driving this synchrony and give examples of how breeding parameters fluctuate across populations and species.

Line 68: In Figure 1 legend the authors could explain how data were simulated in this example.

Lines 73-74: Fecundity is defined as number of young produced per female (see Nagy and Holmes 2004, Journal of Avian Biology 35: 487-491) rather than number of fledged female offspring as stated here.

Lines 75-76: Explain how “increased synchrony in fecundity can increase extinction probability

and lessen the potential for demographic rescue”

Line 77: This phrase is confusing, the authors should be clear what they mean here. Intraspecific is defined as “produced, occurring, or existing within a species or between individuals of a single species”, therefore the phrase “intraspecific passerine populations” doesn't make much sense.

Lines 87-94: This last paragraph attempts to explain why this study was carried out, the specific aims of the project, and how the study contributes to ecology and conservation. I think the authors could make this section clearer - the aims are not well explained and it is not clear how they relate to the text above. What is meant by “two spatial scales” and why is it important? Why did the authors choose these breeding parameters? These are questions that should be addressed here.

Line107: “allowing us to assess breeding parameter synchrony across a large spatial scale.” Explain why is this important.

Line 111: This is a large area. You might expect populations closer together to exhibit more synchronous breeding behaviour compared with species further apart in this range. Did the authors test this hypothesis? This appear to be the case according to text below, but is not made clear in the study objectives in the Introduction.

Line 114: Perhaps the authors could colour points to show number of nests available per year in Figure 2.

Line 122: “MBJV ecoregions were defined using avian ecological data”. Describe how this was done and how this method differs from EPA Level II ecoregions.

Line 126: It looks from Figure 2 that there would have been enough data for analysis in the Appalachian mountain region. The authors could explain here why this was not possible, even for a smaller number of years.

Line 131: Report how many nests per area/year/species contained errors.

Line 132: More often “percentage of eggs which hatch in a clutch” is used to define hatching success. Not clear how the authors calculated the probability that at least one egg hatched. If this is a metric calculated from data, why would a probability be needed? Wouldn't proportion/percentage be correct?

Line 133: What is meant by a “successful” nest?

Lines 140-141: This would reduce the size of your dataset unnecessarily. Perhaps accounting for differences in sample sizes in your statistical analysis would have preserved sample size in this case.

Table 1: Define what is meant by “relative variance” in the table heading. Also, some numbers are overlapping in Table 1

Line 148: It isn't clear here what the authors have done. What was the GLMM used for? What response and explanatory variables were included in the model and what was the reason for doing this? If the purpose is to compare breeding parameters between species/area/year these variables should have been included as explanatory variables. It isn't clear what is meant by “estimated annual parameters” given that in lines 131-133 it is stated that these were calculated from the data.

Line 151: How can you compare a linear mixed model in which one of the models included no random effect? Presumably a different model was used - state here what this was.

Lines 155-156: How many years of data were available? State here.

Line 158: Explain what these linear models were and how they were used in this example.

Line 173: This is a short time series over which to determine whether there is a correlation. Did the authors check to see whether there were enough data points to determine whether there was a significant correlation?

Line 175: Explain why these window sizes were used.

Line 184: Report numbers of nests for each species/area/year here.

Line 187: It is not clear where the results of the GLMMs are in the Results section. What was the purpose of the GLMMs if the results are not reported?

Line 189: There is no record of values/number of nest per year per species/area. Yearly estimates would be helpful in Table 1.

Line 206-243: It is not clear what the importance of these findings are biologically. This study simply checks for synchrony in breeding parameters in different time periods, but it seems as if the authors are searching for significance, with little thought of why one might expect relationships to exist.

Line 250: More explanation could be provided on the importance of examining synchrony in breeding parameters between species, i.e. how this information is of interest biologically or in terms of conservation/species management. Although this study may have identified synchronous fluctuations in breeding parameters between species over time, it is not clear why this is important, particularly as it was only identified in two species. Determining the environmental cause of such synchrony would be interesting, particularly in relation to environmental/climatic changes.

Line 259: Explain why authors think this is a spurious effect.

Line 259: The authors mention the environmental canalisation hypothesis here, but little explanation is given as to what this is and how it applies to this study.

Line 260: Not clear what is meant by “predator guards”. This is more likely to be a local-level effect and is unlikely to affect synchrony in breeding parameters over larger spatial scales.

Line 264: It is not clear why these window sizes were used and whether there was any biological basis for hypothesising that synchrony patterns would be observed at these scales.

Line 268: What evidence is there that synchrony for hatchability has increased in last 20 years?

Line 275: This sentence is highly conjectural, and more clarity is needed in regard to buffering of breeding parameters.

Line 277: A high proportion of ringers (banders) are “citizen scientists” (i.e. they are amateur ornithologists who carry out ringing activities in their spare time and the data are used in scientific studies). Therefore, according to the definition of a “citizen scientist” as a non-professional scientist, ringers/banders are very much in this category.

Line 278: Nevertheless, with appropriate statistical methods, these data are extremely useful for estimating survival (e.g. Freeman and Morgan 1992)

Line 303: It was be useful here to have some description of potential barriers affecting collection of data on some species (e.g. difficulty in accessing nests of some species, local nature protection laws, rarity of species in local areas) and how these barriers may be overcome.
