# Supplementary material for: Using citizen science to determine if songbird nesting parameters fluctuate in synchrony
Source: PLoS One. 2022 Nov 16;17(11):e0277656. doi: 10.1371/journal.pone.0277656 (PMC9668184; doi:10.1371/journal.pone.0277656)
Supplement: Attachment — Submitted filename: R2 Response to Reviewers_final.docx [file pone.0277656.s005.docx]

Reviewer’s Comments

**Reviewer 2**

Thank you for taking the time to review our revised manuscript and providing your helpful comments. We incorporated your suggestions to clarify our language and conducted a careful read-through to remove typos. We also provided the statistical results of our synchronous trends as suggested in comment 24. Finally, we addressed your other suggestions. Please note that line numbers in your comments and our responses refer to those in the document with track changes (Revised Manuscript with Track Changes_final.docx).

1. *L24: I suggest deleting the phrase “inter- and intraspecific”. Or else changing to “inter- and intraspecific interactions among wildlife populations”. Otherwise, the meaning is unclear.*
   a. We agree that the meaning behind this original phrasing is unclear and specified that the referenced implications are for interactions among and within wildlife populations.
2. *L28: typo: double comma*
   a. Thank you for pointing this out. We have removed the extra comma.
3. *L40: “…study species may be resilient to climate change”: Can you insert somewhere earlier in the abstract that weather/climate is a common driver of spatial synchrony in population dynamics? That would make this statement seem more believable, connecting the dots between climate change and population synchrony.*
   a. That’s a great suggestion! We added a statement in lines 24–25 noting that variations in climate are a common drive of spatial synchrony.
4. *L49-50: This sentence requires refinement, perhaps as follows: “Studies focusing on sub-populations and smaller regions are common, but are inadequate for understanding population dynamics across a species' range.”*
   a. Thank you for this suggestion. We feel that this revised sentence is more concise and accurately reflects the intent of the original statement.
5. *L54: typo: double period after “United States declined [5]”*
   a. We removed the extra period.
6. *L55-58: Most widespread populations exhibit both areas of high and low spatial synchrony within populations (e.g., Allen et al. “Mapping spatial synchrony…”, Conservation Bio, 2021). I suggest revising to something like the following: “...likely vary among populations and ecoregions, but we also know that common environmental changes experienced by nearby populations can induce synchronous fluctuations in their demographic parameters." Maybe cite Schaub paper at the end?*
   a. This is a great suggestion that clarifies our original statement! We made the suggested revisions and cited Schaub et al. (2015).
7. *L66: The Moran effect is a special case of environmental synchronization, so I would rephrase “dispersal and the Moran effect” to “dispersal and environmental effects, including the Moran effect, which proposes …”*
   a. We specified that the Moran effect is one type of environmental synchronization.
8. *L78 (Fig 1. Legend): Please specify: synchrony of what? (Some demographic parameter.)*
   a. Thank you for pointing this out. We specified the caption that the scenario depicts various fluctuations in a given demographic parameter.
9. *L87-88: Suggest shortening: "two intraspecific passerine populations (i.e., two populations of the same species)" to "two populations of the same passerine species".*
   a. For brevity, we incorporated your suggested wording into this sentence.
10. *L96: Suggest changing “spatially large scales” to “large spatial scales”.*
    a. We incorporated your suggested wording into this sentence.
11. *L107: here you say “few studies” but I thought in the Abstract you say that none had?*
    a. We apologize for this discrepancy. When we drafted our original manuscript, we were unaware that other studies had used citizen science data to assess if demographic parameters fluctuated in synchrony. Another reviewer pointed out examples of these studies, and we revised the statement in the Introduction but forgot to do so in the Abstract. We modified the Abstract (line 28) to state that a few studies have studied this phenomenon.
12. *L152-155: Can you make it clear that the 7 MBJV ecoregions are smaller units that fall within the broader EPA ecoregion II? And can you avoid using the word “ecoregions” for both the EPA and the MBJV units? Perhaps you can refer to the EPA units as “regions”. Further, I think it may be clearer to list only the 2 MBJV ecoregions that you did include and simply describe why you excluded others without naming them.*
    a. MBJV ecoregions are not always smaller than EPA ecoregions, and their boundaries do not always match those of EPA ecoregions. For example, the MBJV’s Atlantic Coast ecoregion encompasses the entirety of Florida and Maine, whereas under the EPA Level II model, these states contain three and two ecoregions, respectively.
    b. We agree using the term “ecoregions” for the MBJV and EPA units is confusing. Because “ecoregions” is the proper term for the EPA units, and to avoid confusing readers who are familiar with the EPA model, we replaced the word “ecoregion” with “region” when referring to the MBJV model. We also removed the unused MBJV regions from lines 154–155 and stated in lines 160–162 that we considered an additional five regions but excluded them because of lack of data.
13. *L156: The line about EPA Level III and IV should appear immediately after the sentence stating that you restricted the study to EPA Level II. I suggest rewording it as follows: “EPA Level III and IV ecoregions were not included as they are smaller…”*
    a. We apologize for the misunderstanding, but we did not use EPA Level II ecoregions in our study. To avoid confusion, we clarified this in the text (line 156) and stated our reasons why we used the MBJV regions instead (lines 156–160).
14. *L172: change “considered” to “calculated”*
    a. We made the necessary correction.
15. *L175: Should this read: “The only nesting parameter not available…” instead? Also, is it really the “only” one? I suggest changing that to a simple statement saying that clutch size was not available.*
    a. We apologize for the confusion of our original wording. We meant to state that although clutch size data were available, we did not consider this parameter because it typically varies more between the first and last clutch of a season for bluebirds than it does between years. We rephrased the sentence to read “The only nesting parameter that we did not consider was clutch size…”
16. *L177-179: I suggest deleting: “We estimated parameters for AC bluebirds and chickadees, and CH bluebirds and chickadees.” And replacing it with “We estimated each parameter separately for each species and ecoregion.” Or else including that information elsewhere.*
    a. We apologize if our original wording was not clear. We cannot make the suggestion as is because although we estimated nesting parameters for each species, we did not estimate these for each region. We revised the sentence to state that we estimated nesting parameters for each species and each species-region combination.
17. *Table 1: I don’t think “Range” is the appropriate name for a column that describes “number of nests”. Maybe “n per year” would be more appropriate?*
    a. We apologize for the confusion. The values in the column “Range” reflect the minimum and maximum number of nests used in analyses per year, as the number of observations were not consistent across time. We changed the caption of Table 1 (line 180) to clarify what these values refer to.
18. *L194-195: “and this led to unrealistic annual parameter estimates (e.g., 100% hatchability every year).” It isn’t clear to me why a longer nest-check interval would lead to estimates of 100% hatchability every year. I suggest you simply explain why you used a simpler method (simple GLM vs. logistic exposure GLM) based on the data limitations you describe (i.e., a long check interval isn’t recommended for that technique, a point which could be reinforced by citing the original Shafer 2004 paper). You could also state in that sentence why you believe the resulting (upward) bias in estimates is not a major concern. That is, because it is likely consistent across regions and species.*
    a. We modified our statement to specify that the logistic-exposure method recommends nest check intervals of 3–5 days, and our nest intervals spanned a larger range (lines 192–194).
    b. Regarding the statement that the upward bias in our estimates is not a concern, we discussed this in lines 197–199.
19. *L200-202: I suggest deleting the sentence “The model with nest location…”. You can include such a justification in the Table footnotes perhaps? But I don’t think it is necessary.*
    a. We removed this sentence from the Methods and added it to the caption of S1 Table 1 (lines 558-560).
20. *L207: The first mention of the word “canalized” is unclear without a definition. I suggest replacing the first mention with the actual meaning of the word. Then you can explain that this is part of the environmental canalization hypothesis.*
    a. Thank you for this suggestion. To make the meaning clearer to readers, we added a short definition of the term (lines 206–207) before referring to the term itself.
21. *L218: “closer to one” should be “closer to or above one” as (at least CV) can exceed 1 if variability is very high.*
    a. We added a statement noting that values of or greater than one are possible for CV and indicate high variability.
22. *L245-249: Minor point, but I think it would be just as easy (and maybe more meaningful) to simply state how many nests of each species occurred in each region (4 numbers total).*
    a. Thank you for the suggestion. We added the number of nests found at the species and region levels.
23. *L266: typo: “specie” should be “species”*
    a. We changed “specie” to “species”.
24. *L286-288: “However, we detected increasing trends in positive correlations…” I think this is a most interesting result and provides a lot of “food for thought” for potential causes the Discussion. Can you provide statistical evidence for the trends? Slope estimate and CI or p value? If “significant”, it seems like this is a real result that contradicts statements elsewhere that no real patterns were observed.*
    a. This is an excellent suggestion! We included synchrony trend methods and results using linear models of correlation coefficients against time (i.e., sliding windows; lines 241–243 and lines 275–313).
    b. Though some of these trends were significant and showed increasing or decreasing patterns in synchrony, this does not contradict other statements (lines 35, 332, and 354) that we did not observe consistent patterns across parameters, species, and regions.
25. *L342: I don’t think you can state “potentially a spurious effect” here, parenthetically, without providing evidence or reasoning.*
    a. We removed this phrase.
26. *L352: can you remind the reader why it was not expected? Is this because of the canalization hypothesis?*
    a. This is an excellent suggestion! We explained that we expected to find little synchronization because nesting parameters for short-lived and highly productive passerines are typically canalized against temporal variability, and therefore would be expected to show little synchrony as well (lines 352–353).
27. *L359-360: “synchrony is occurring” This language strikes me as unnecessarily binary. The synchrony may be too weak to detect.*
    a. The synchrony may indeed be too weak to detect, but because we only found one parameter with a significant overall correlation, we cannot definitively state that synchrony is or isn’t occurring. We added a statement specifying that if synchrony is present, we cannot detect it.
28. *L373: “this one negative trend toward increased synchrony” … please explain again to what negative trend you are referring here. It was introduced in the previous paragraph and “this” is ambiguous. Also, how do you reconcile this statement with your discussion of a positive trend in hatchability synchrony above. Can you be more precise or consistent with your language regarding these “trends”?*
    a. In light of our new synchrony trend analyses per the suggestion in comment 24, we reworded this part of the Discussion to include our new results (lines 334–338, 369–373).
29. *Figure 3. The labels across the top (i.e., the parameters) are very helpful. Similar labels for the species/region across the left side are needed to make the figure easy to interpret without frequently referring to the legend.*
    a. Thank you for this suggestion. We added labels for each level of species and region to make it easier for readers to view our results.
